# Supplementary figures and images for: Dissemination of local sub-variants of SARS-CoV-2 detected by detailed mutation analysis in wastewater-based epidemiology
Source: PLoS One. 2025 May 28;20(5):e0317076. doi: 10.1371/journal.pone.0317076 (PMC12118838; doi:10.1371/journal.pone.0317076)

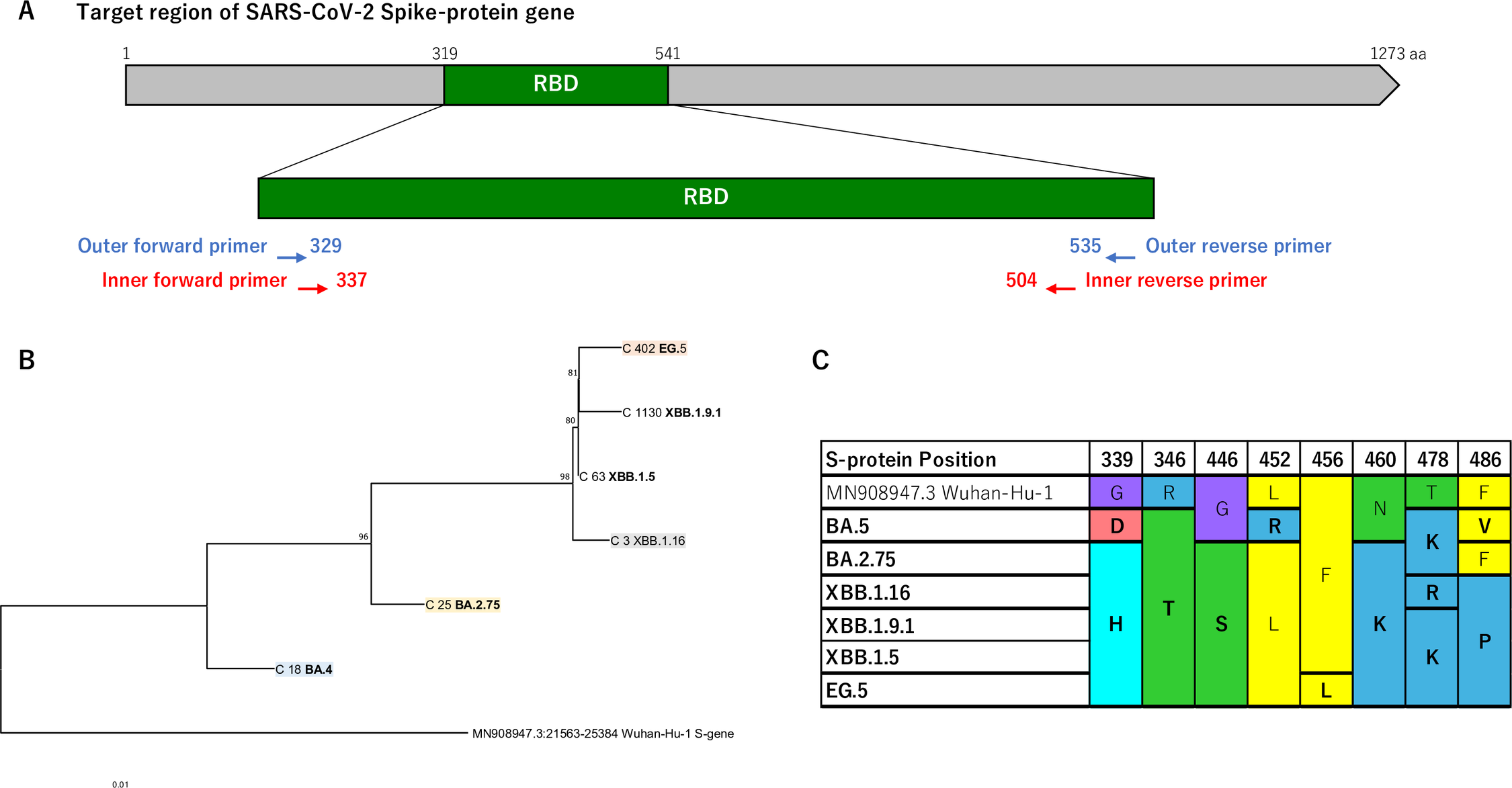

Supplement: S1 Fig — (A) The target region of the S gene. (B) Phylogenetic tree of the major variants. (C) mutations of the major variants in the target region. (TIF) [file pone.0317076.s002.tif]

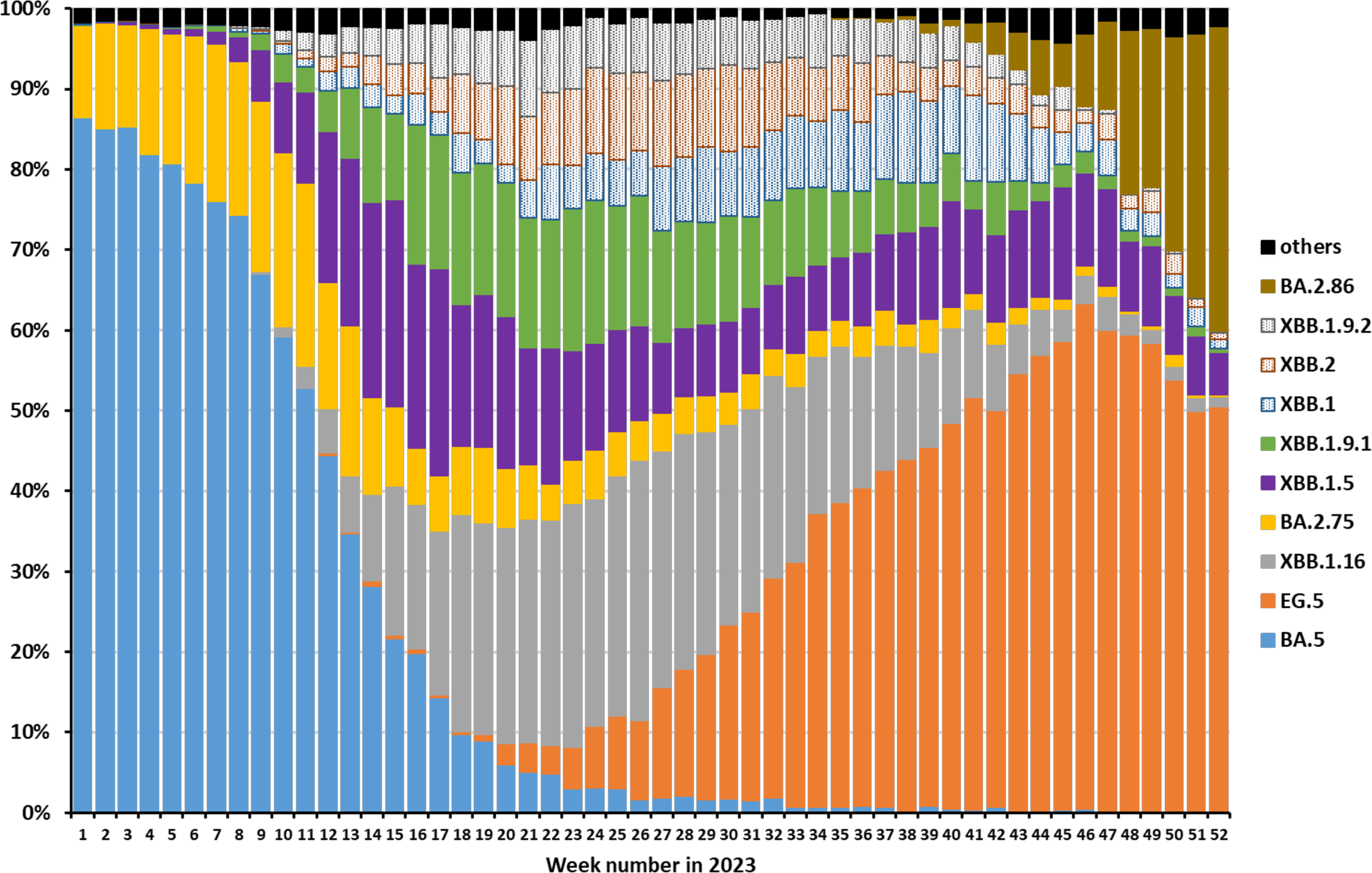

Supplement: S2 Fig — (TIF) [file pone.0317076.s003.tif]

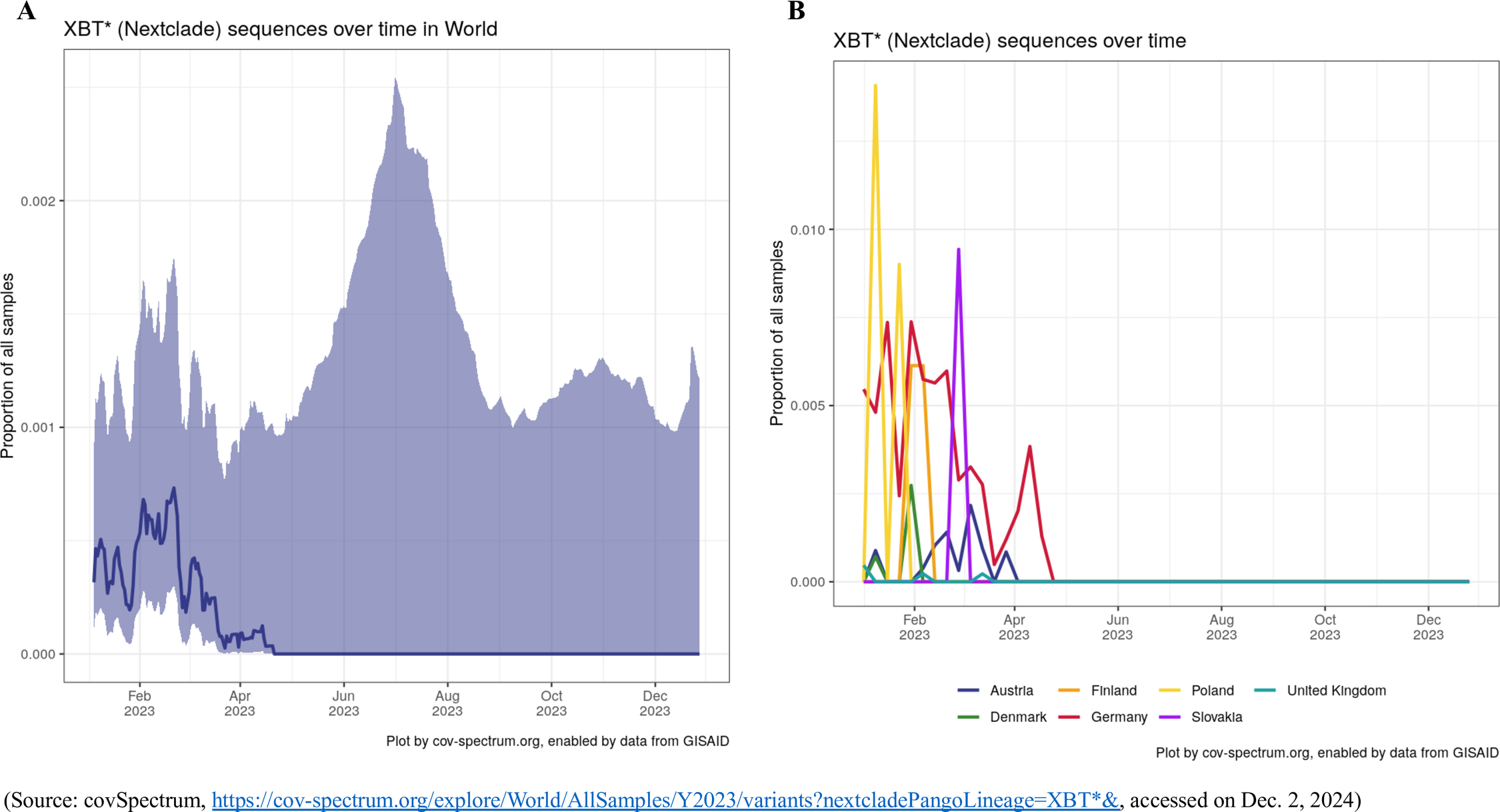

Supplement: S3 Fig — (A) Proportion in the world. (B) Proportion by country. (TIF) [file pone.0317076.s004.tif]

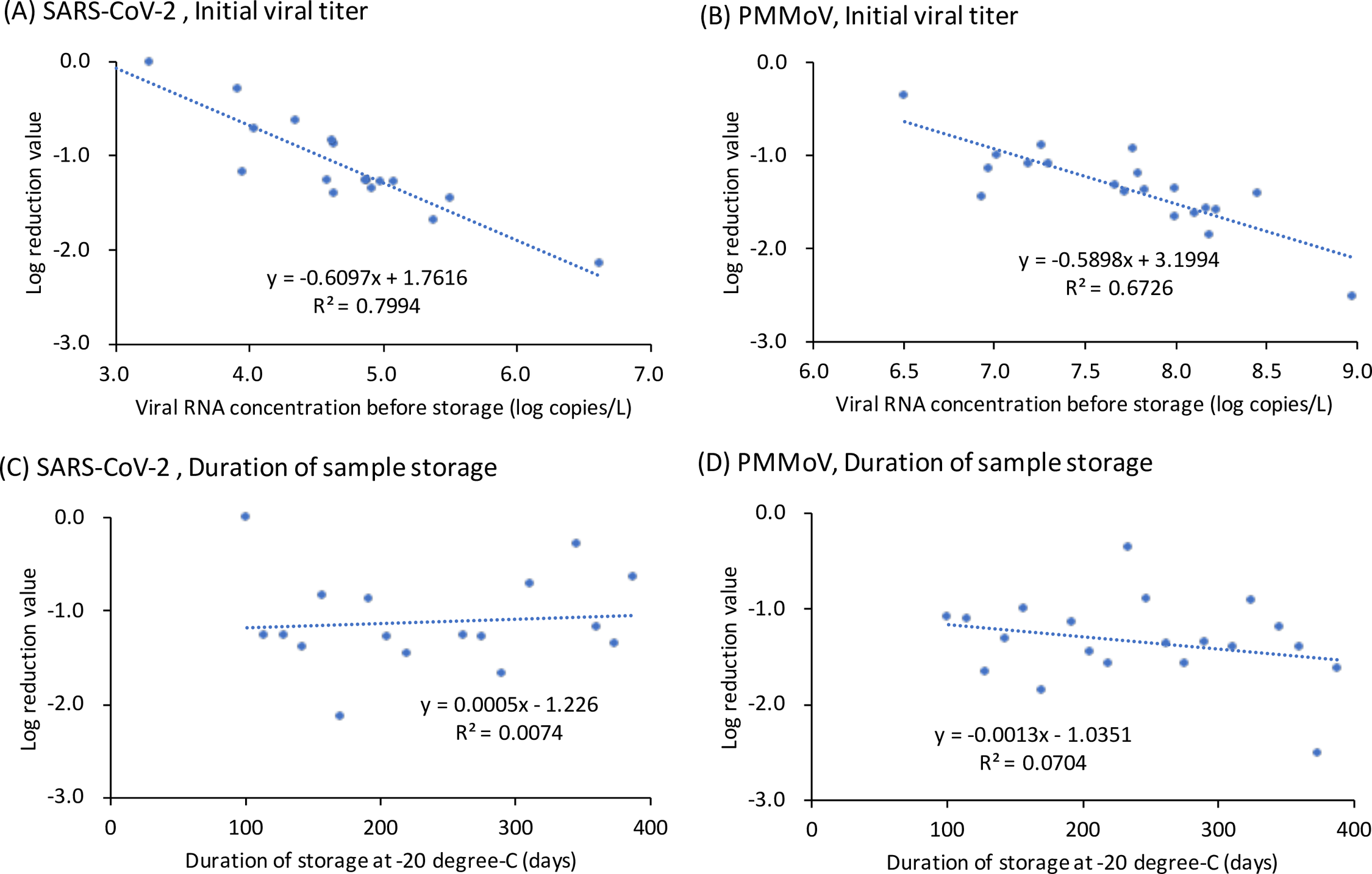

Supplement: S4 Fig — (A) Log reduction of SARS-CoV-2 by the initial concentration. (B) Log reduction of PMMoV by the initial concentration. (C) Log reduction of SARS-CoV-2 by duration of storage. (D) Log reduction of PMMoV by duration of storage. The SARS-CoV-2 RNA concentrations were quantified with CDCN1 assay. (TIF) [file pone.0317076.s005.tif]
