# Supplementary material for: Dissemination of local sub-variants of SARS-CoV-2 detected by detailed mutation analysis in wastewater-based epidemiology
Source: PLoS One. 2025 May 28;20(5):e0317076. doi: 10.1371/journal.pone.0317076 (PMC12118838; doi:10.1371/journal.pone.0317076)
Supplement: Attachment — Submitted filename: Responses Letter PONE-S-24-7647R1_RC20250408.docx [file pone.0317076.s011.docx]

**Responses to the reviewer comments for PONE-S-24-76473**

We do appreciate all reviewers for sharing their precious time to review our manuscript and for valuable comments to improve it. Here are our responses to the comments and revisions made in the manuscript.

**Reviewer #1:**

*The study highlights the potential of wastewater-based epidemiology (WBE) to go beyond merely quantifying variants by tracing the emergence and transmission pathways of SARS-CoV-2 variants. This is interesting work; here my suggestions.*

1. *The introduction lacks necessary citations to ensure that all previous work is cited properly. Some references need to be updated to represent the literature accurately. For example, this sentence does not have the necessary citation diversity, and recent ones, “Several studies have successfully detected and identified SARS-CoV-2 variants in wastewater (Kuroiwa et al., 2023; Jahn et al., 2022; Layton et al., 2022).” Several recent studies identified the new variants through WBE, such as BA 2.86 and JN 1; therefore, these need to be cited because they lay the foundation for identifying SARS-CoV-2 new variants in WBE.*

Response:

We appreciate for your comments. We have reviewed the suggested citations and added some more citations.

**L. 25-26**

Several studies have successfully detected and identified SARS-CoV-2 variants in wastewater [2–7]

**L.39-42**

In contrast, SARS-CoV-2 genome sequences obtained from wastewater usually comprise only partial genome regions (e.g., the spike-protein region) or an array of multiple partial regions across the entire genome, making it difficult to assemble the entire genome and identify each variant present in a wastewater sample [2–5].

**References**

2. Jahn, Katharina, David Dreifuss, Ivan Topolsky, Anina Kull, Pravin Ganesanandamoorthy, Xavier Fernandez-Cassi, Carola Bänziger, et al. 2022. “Early Detection and Surveillance of SARS-CoV-2 Genomic Variants in Wastewater Using COJAC.” Nature Microbiology 7 (8): 1151–60. https://doi.org/10.1038/s41564-022-01185-x.
3. Layton, Blythe A, Devrim Kaya, Christine Kelly, Kenneth J Williamson, Dana Alegre, Silke M Bachhuber, Peter G Banwarth, et al. 2022. “Evaluation of a Wastewater-Based Epidemiological Approach to Estimate the Prevalence of SARS-CoV-2 Infections and the Detection of Viral Variants in Disparate Oregon Communities at City and Neighborhood Scales.” Environmental Health Perspectives 130 (6): 1–15. <https://doi.org/10.1289/EHP10289>.
4. Iwamoto, Ryo, Kiyoshi Yamaguchi, Kotoe Katayama, Hiroki Ando, Ken ichi Setsukinai, Hiroyuki Kobayashi, Satoshi Okabe, Seiya Imoto, and Masaaki Kitajima. 2023. “Identification of SARS-CoV-2 Variants in Wastewater Using Targeted Amplicon Sequencing during a Low COVID-19 Prevalence Period in Japan.” Science of the Total Environment 887 (August). <https://doi.org/10.1016/j.scitotenv.2023.163706>.
5. Kuroiwa, Miho, Yoshinari Gahara, Hirohito Kato, Yuji Morikawa, Yuki Matsui, Takumi Adachi, Shin Kurosawa, Tomohiro Kuroita, Yoshinori Ando, and Masatomo Rokushima. 2023. “Targeted Amplicon Sequencing of Wastewater Samples for Detecting SARS-CoV-2 Variants with High Sensitivity and Resolution.” Science of the Total Environment 893 (June): 164766. https://doi.org/10.1016/j.scitotenv.2023.164766.
6. Carmen Espinosa-Gongora, Carlo Berg, Moa Rehn, Javier Edo Varg, Lena Dillner, Neus Latorre-Margalef, Anna J Székely, Emmi Andersson, Elin Movert. 2023. “Early detection of the emerging SARS-CoV-2 BA.2.86 lineage through integrated genomic surveillance of wastewater and COVID-19 cases in Sweden, weeks 31 to 38 2023.” Euro Surveillance Nov;28(46):2300595. https://doi: 10.2807/1560-7917.
7. Vinay Rajput, Rinka Pramanik, Kiran Nannaware, Vinita Malik, Sejal Matra, Shubham Kumar, Sai Joshi, Pradnya Kadam, Unnati Bhalerao, Manisha Tupekar, Dipti Deshpande, Priyanki Shah, Poornima Sangewar, Niharika Gogate, Radhika Boargaonkar, Dhawal Patil, Saurabh Kale, Asim Bhalerao, Nidhi Jain, L S Shashidhara, Sanjay Kamble, Syed Dastager, Krishanpal Karmodiya, Mahesh Dharne. 2024. “Wastewater surveillance in post-omicron silent phase uncovers silent waves and cryptic transmission of SARS-CoV-2 variants; a yearlong study in Western India.” Science of the Total Environment 955 (Dec): 176833. https://doi: 10.1016/j.scitotenv.2024.176833.
8. *Also, lines 56 and 59, 68 to 71 need appropriate references added.*

Response:

We appreciate for your comments. We have added relevant citations.

**L.29-31**

Clinical genomic surveillance typically focuses on a limited number of selected clinical samples, potentially overlooking variants present in the broader population [3].

**L.33-36**

However, the variant sequences obtained via wastewater genomic surveillance are heterogeneous and unsuitable for assembly into a complete genome sequence, which is essential for accurate variant lineage identification [2–3].

**References**

2.	Jahn, Katharina, David Dreifuss, Ivan Topolsky, Anina Kull, Pravin Ganesanandamoorthy, Xavier Fernandez-Cassi, Carola Bänziger, et al. 2022. “Early Detection and Surveillance of SARS-CoV-2 Genomic Variants in Wastewater Using COJAC.” Nature Microbiology 7 (8): 1151–60. https://doi.org/10.1038/s41564-022-01185-x.

3.	Layton, Blythe A, Devrim Kaya, Christine Kelly, Kenneth J Williamson, Dana Alegre, Silke M Bachhuber, Peter G Banwarth, et al. 2022. “Evaluation of a Wastewater-Based Epidemiological Approach to Estimate the Prevalence of SARS-CoV-2 Infections and the Detection of Viral Variants in Disparate Oregon Communities at City and Neighborhood Scales.” Environmental Health Perspectives 130 (6): 1–15. https://doi.org/10.1289/EHP10289.

9. *The study discussion is minimal and needs to be revised. For example, EG.5 sub-variant C65601, XBB.1.16 sub-variant C65598, and BA.2.75 sub-variant C676 were detected only in Komatsu this can be due to WBE coverage differences in two cities 40% of Komatsu’s population and 57% of Hamamatsu’s ? these needs to be discussed.*

Response:

We appreciate for your valuable comment. Since WBE can target only population in the sewer catchment (i.e., sewershed), it is unknown whether these sub-variants were present in outside of the sewer catchment of the target cities. Strictly speaking, our discussion focuses on the sewer catchment of each target city, not on the entire city. Moreover, some sub-variants might not be detected due to sampling bias and sequencing failure. Sampling bias might occur due to grab sampling, where samples were collected only at the morning peak-flow time. By the sequencing failure, sequence data was successfully acquired from only 8/18 of the samples particularly in Hamamatsu. These limitations are explained in detail in the response to the below comment #1-6 and have been described in the revised manuscript.

Nevertheless, emergence of these local sub-variants was also supported by comparison with clinical variants data in GISAID. Most of the detected local sub-variants were phylogenetically close to the predominant clinical variants in Japan reported in GISAID. According to the phylogenetic analysis shown in Fig. 2, the EG.5 sub-variant C65601 was diverged from C402, which were the most predominant EG.5 strain in Japan. Similarly, BA.2.75 sub-variants C676, C661, and C57075 are indicated to evolved from the same origin of C25, which were the first and second most predominant BA.2.75 strains in Japan, respectively.

In summary, though it is hard to conclude absence of these sub-variants in the other city, the haplotype analyses demonstrate that predominant strains in each target sewershed consisted of multiple haplotypes rather than a single sub-variant, suggesting emergence of unique sub-variants driven by local evolutionary processes.

We added this discussion in the revised manuscript as below. The limitations in methodology were also added in Implications and limitations section.

**L.223-234**

Although the absence of these sub-variants in the other city might be caused by sampling bias and sequencing failure, emergence of the local sub-variants was also supported by comparison with clinical variant data reported in the GISAID database. Most of the detected local sub-variants were phylogenetically close to the predominant clinical variants in Japan. According to the phylogenetic analysis (Fig 2), the EG.5 sub-variant C65601 was diverged from C402, which were the most predominant EG.5 strain in Japan. Similarly, BA.2.75 sub-variants C676, C661, and C57075 are indicated to evolved from the same origin of C25, which were the most predominant BA.2.75 variant in Japan, respectively. These results demonstrate that predominant strains in each target sewershed consisted of multiple sub-variants rather than a single sub-variant, suggesting emergence of unique sub-variants driven by local evolutionary processes.

10. *The duration differences for variant prevalence between cities (e.g., 35 weeks in Komatsu versus 1 week in Hamamatsu) are interesting but require more in-depth discussion. This can be due to population diversity between cities or other factors that need to be discussed.*

Response:

This is an important question although it is not easy to guess reasons. Difference of epidemic duration by variants and regions were also reported in clinical variant data of Japan (Nakagawa et al. 2023). These sub-variants detected in wastewater were phylogenetically close to the clinically predominant variants in the prefecture where the target city is located, and their epidemic durations were similar to those of the clinically predominant variants.

According to the clinical variant data in GISAID, BA.2.75 was reported until 25th week in in Ishikawa Pref, where Komatsu City is located, while it was continuously reported until 12th week in Shizuoka Pref., where Hamamatsu City is located. Similarly, BA.5 was reported continuously in Ishikawa Pref. until the 22nd week, while it was not reported after the 13th week in Shizuoka Pref. is located (S4 Table). In Japan, mechanistic reasons for regional differences of epidemic variants are probably due to human mobility and public health policies, rather than genetic diversity of population (Konishi, 2021). Particularly, migration of a variant with high infectivity from another city would enhance replacement of predominant variants (Nakagawa et al. 2023).

An important finding of this study is that application of the haplotype analysis to WBE enables to identify regional differences in epidemic durations of variant at the city level, with a geographically higher resolution than at the prefecture level.

We added this discussion in the revised manuscript as below.

**L.188-201**

Such difference of epidemic duration by variants and regions were also reported in clinical data of Japan [9]. According to the clinical variant data in GISAID, BA.2.75 was reported until 25th week in in Ishikawa Pref, where Komatsu City is located, while it was continuously reported until 12th week in Shizuoka Pref., where Hamamatsu City is located (S4 Table). In Japan, regional differences of epidemic variants are probably caused by human mobility and public health policies. Particularly, migration of a variant with high infectivity from another city would facilitate replacement of predominant variants [26]. In this study, the sub-variants detected in wastewater were phylogenetically close to the clinically predominant variants in the prefecture where the target city is located, and their epidemic durations were similar to those of the clinically predominant variants (S2 Table). These results suggest that applying detailed mutation analysis to WBE enabled the identification of regional differences in epidemic durations by variant at the city level, with a geographically higher resolution than at the prefecture level.

**S4 Table. Number of clinical sub-variants reported in GISAID in the whole Japan, Ishikawa Prefecture, and Shizuoka Prefectur**e.

*1: Japan is the number of sequences in all other areas excluded Ishikawa Pref., Shizuoka Pref., and Hamamatsu City.

*2: Ishikawa Pref. includes Komatsu City.

*3: Shizuoka Pref. includes Hamamatsu City.

*4: 2023-W1 indicates the first week of 2023.

**Reference**

26.	Konishi, Tomokazu. 2021. “Effect of Control Measures on the Pattern of COVID-19 Epidemics in Japan.” PeerJ 9 (September). https://doi.org/10.7717/peerj.12215.

11. *While the findings emphasize WBE's utility, the connection between wastewater data and real-world epidemiological trends is less discussed regarding variant landscape; for example, “some sub-variants that were not reported in clinical specimens were detected in wastewater," but there is no discussion of how these WBE findings are essential in terms of clinical utility or public health.*

Response:

The primary purpose of this scientific study is to demonstrate that application of haplotype analysis to wastewater genomic surveillance enables to clarify regional dissemination pathway and local evolutionary processes of variants. One important benefit in application of the haplotype analysis to wastewater genomic surveillance is to identify mutations in a population more comprehensively than clinical genomic surveillance. For example, nonsense mutations such as frameshifts and stop codons were detected in this study as well as a previous study on wastewater genomic surveillance (Napit et al. 2023). Such nonsense mutations are hardly identified by clinical genomic surveillance, which targets samples from symptomatic patients. This study demonstrated that the haplotype analysis of wastewater data could reveal virus’s evolutionary trajectory, including the emergence of nonsense mutations like frameshifts and stop codons, which would be overlooked by clinical genomic surveillance.

Although this study does not primarily aim to prove the direct utility to public health polices, we assume these analyses is potentially applied to public health policies, e.g., early warning on emergence of a new variant.

In general, benefits of wastewater genomic surveillance compared to clinical genomic surveillance include:

- More efficient and frequent surveillance (e.g., on a weekly basis) due to the smaller number of samples required.
- Better representativeness of the actual proportions of variants in a target area, as it is less affected by sampling biases.
- A lower likelihood of overlooking epidemic variants due to reduced sampling biases.

In addition to these general benefits, the application of haplotype analysis would enable:

- Early detection of the emergence of local variants.
- Identification of the origins and dissemination pathways at both national and regional levels.
- Insights into the evolutionary processes of the virus, including the emergence of asymptomatic variants.

We added this discussion in the revised manuscript as below.

**L.262-272**

Detection of nonsense mutations, such as frameshifts and stop codons, in SARS-CoV-2 genomic sequences in wastewater has been reported in a previous study [30]. However, such nonsense mutations are rarely identified by clinical genomic surveillance because clinical genomic surveillance primarily relies on samples from symptomatic and diagnosed patients only. In this study, we demonstrate for the first time the potential for elucidating the mechanisms driving the transition of epidemic lineages due to the emergence of frameshifts and stop codons through nucleotide-level monitoring of Omicron sub-lineages. applying haplotype analysis to wastewater-based epidemiology (WBE) enables effective detection of these mutations, providing insights into more detailed evolutionary trajectories of the virus.

**L.277-297**

Compared to clinical genomic surveillance, wastewater genomic surveillance offers several advantages: (i) more efficient and frequent surveillance (e.g., on a weekly basis) due to the smaller number of samples required; (ii) better representativeness of the actual proportions of variants in a target area; and (iii) a lower likelihood of overlooking epidemic variants. These benefits complement clinical genomic surveillance, which requires a large number of samples and is subject to significant biases in sample selection. Recent studies reported that wastewater genomic surveillance could detect emerging variants earlier than through clinical surveillance [5, 31–32]. Moreover, deep sequencing of the RBD region detected cryptic SARS-CoV-2 lineages that were not found in any global databases [33]. The findings of this study also demonstrate that the utility of wastewater genomic surveillance for detection of SARS-CoV-2 sub-variants that have been uncovered by clinical surveillance. Furthermore, detailed mutation analysis in wastewater genomic surveillance facilitates the early detection of emerging local variants and the identification of their origins and dissemination pathways by comparing them with those in other countries and regions. In this study, haplotype analysis was applied to a short region within the RBD of the S gene. However, recent advancements in sequencing techniques have enabled the analysis of whole genome sequences of SARS-CoV-2 from wastewater [34–36]. Applying haplotype analysis to whole-genome sequences obtained from wastewater is expected to provide a more comprehensive view of mutation trajectories, enhancing our understanding of viral evolution, including the emergence of nonsense mutations and asymptomatic variants.

**Reference**

30.	Napit, Rajindra, Prajwol Manandhar, Ashok Chaudhary, Bishwo Shrestha, Ajit Poudel, Roji Raut, Saman Pradhan, et al. 2023. “Rapid Genomic Surveillance of SARS-CoV-2 in a Dense Urban Community of Kathmandu Valley Using Sewage Samples.” PLoS ONE 18 (3 March). <https://doi.org/10.1371/journal.pone.0283664>.

31.	Karthikeyan, Smruthi, Joshua I. Levy, Peter de Hoff, Greg Humphrey, Amanda Birmingham, Kristen Jepsen, Sawyer Farmer, et al. 2022. “Wastewater Sequencing Reveals Early Cryptic SARS-CoV-2 Variant Transmission.” Nature 609 (7925): 101–8. https://doi.org/10.1038/s41586-022-05049-6.

32.	La Rosa, G., D. Brandtner, G. Bonanno Ferraro, C. Veneri, P. Mancini, M. Iaconelli, L. Lucentini, et al. 2023. “Wastewater Surveillance of SARS-CoV-2 Variants in October–November 2022 in Italy: Detection of XBB.1, BA.2.75 and Rapid Spread of the BQ.1 Lineage.” Science of the Total Environment 873 (May). https://doi.org/10.1016/j.scitotenv.2023.162339.

33.	Smyth, Davida S., Monica Trujillo, Devon A. Gregory, Kristen Cheung, Anna Gao, Maddie Graham, Yue Guan, et al. 2022. “Tracking Cryptic SARS-CoV-2 Lineages Detected in NYC Wastewater.” Nature Communications 13 (1). https://doi.org/10.1038/s41467-022-28246-3.

34.	Barbé, Laure, Julien Schaeffer, Alban Besnard, Sarah Jousse, Sébastien Wurtzer, Laurent Moulin, Françoise S. le Guyader, and Marion Desdouits. 2022. “SARS-CoV-2 Whole-Genome Sequencing Using Oxford Nanopore Technology for Variant Monitoring in Wastewaters.” Frontiers in Microbiology 13 (June). https://doi.org/10.3389/fmicb.2022.889811.

35.	Child, Harry T., Paul A. O’Neill, Karen Moore, William Rowe, Hubert Denise, David Bass, Matthew J. Wade, et al. 2023. “Optimised Protocol for Monitoring SARS-CoV- 2 in Wastewater Using Reverse Complement PCR-Based Whole-Genome Sequencing.” PLoS ONE 18 (4 APRIL). https://doi.org/10.1371/journal.pone.0284211.

36.	Xu, Xiaoqing, Yu Deng, Jiahui Ding, Xiawan Zheng, Chunxiao Wang, Dou Wang, Lei Liu, et al. 2023. “Wastewater Genomic Sequencing for SARS-CoV-2 Variants Surveillance in Wastewater-Based Epidemiology Applications.” Water Research 244 (October). https://doi.org/10.1016/j.watres.2023.120444.

12. *Acknowledge the study's limitations, such as sampling frequency, geographical scope, and reliance on wastewater data alone.*

Response:

The major limitation of this study was sequencing failure in some samples, which was probably caused by RNA degradation in sample storage and processing, and presence of PCR inhibiting substance in wastewater. In general, wastewater genomic surveillance targets entire population in the sewer catchment, while clinical genomic surveillance targets diagnosed patients. This study demonstrated that the difference of the target population is not a drawback of wastewater genomic surveillance but potentially enables to reveal detailed evolutionary steps of variants by identifying presence of nonsense mutations, which cannot be acquired in clinical surveillance targeting symptomatic patients only. As for the sampling frequency, wastewater genomic surveillance enables to clarify shifts of variants with higher time-resolution by increasing sampling frequency, compared to clinical genomic surveillance.

We created a section to summarized benefits of application of the haplotype analysis to wastewater genomic surveillance and limitations of this study as below.

**L.273-341**

**Implications and limitations**

In summary, wastewater genomic surveillance enables not only the identification of variant trend overlooked by clinical genomic surveillance but also the detection of sub-variants with locally unique genomic shifts through the detailed mutation analysis of amplicon sequences. Compared to clinical genomic surveillance, wastewater genomic surveillance offers several advantages: (i) more efficient and frequent surveillance (e.g., on a weekly basis) due to the smaller number of samples required; (ii) better representativeness of the actual proportions of variants in a target area; and (iii) a lower likelihood of overlooking epidemic variants. These benefits complement clinical genomic surveillance, which requires a large number of samples and is subject to significant biases in sample selection. Recent studies reported that wastewater genomic surveillance could detect emerging variants earlier than through clinical surveillance [5, 31–32]. Moreover, deep sequencing of the RBD region detected cryptic SARS-CoV-2 lineages that were not found in any global databases [33]. The findings of this study also demonstrate that the utility of wastewater genomic surveillance for detection of SARS-CoV-2 sub-variants that have been uncovered by clinical surveillance. Furthermore, detailed mutation analysis in wastewater genomic surveillance facilitates the early detection of emerging local variants and the identification of their origins and dissemination pathways by comparing them with those in other countries and regions. In this study, haplotype analysis was applied to a short region within RBD of the S gene. However, recent advancements in sequencing techniques have enabled the analysis of whole genome sequences of SARS-CoV-2 from wastewater [34–36]. Applying haplotype analysis to whole-genome sequences obtained from wastewater is expected to provide a more comprehensive view of mutation trajectories, enhancing our understanding of viral evolution, including the emergence of nonsense mutations and asymptomatic variants.

Since genetic mutations occur randomly, not all mutations are beneficial for viral replication; they also include deleterious and neutral mutations, the latter of which are neither advantageous nor disadvantageous. Strains carrying deleterious mutations are eliminated through selection, whereas those with neutral mutations persist and contribute to the formation of diverse sub-variants in different regions. In contrast, variants identified in clinical settings primarily possess mutations that enhance human infectivity (i.e., beneficial for replication). Consequently, both clinical and wastewater genomic surveillance that rely on existing catalogs of known clinical variants face challenges in capturing the evolutionary pathways leading to the emergence of novel viral lineages. To address this limitation, this study employed a nucleotide-level mutation analysis, including synonymous substitutions, rather than focusing solely on amino acid changes. This approach enables a more comprehensive understanding of viral variant diversity, particularly neutral mutations, and represents a novel and advantageous method compared to conventional approaches. By incorporating such analyses, we can better elucidate the evolutionary dynamics of the virus and the detailed trajectories leading to the emergence of new clinical variants.

Despite these advantages, several limitations of wastewater genomic surveillance should be noted. The representativeness of wastewater genomic surveillance primarily depends on the sampling methodology. Grab sampling, often conducted during morning peak flow, may fail to detect variants discharged at other times. Composite sampling, which collects multiple samples over a 24-hour period, improves representativeness but may still miss variants released between sampling intervals. Additionally, improving sample storage and processing methods has been suggested to enhance the reliability of mutation analysis in wastewater genomic surveillance. In this study, sequence data was successfully obtained in only 21 of 38 samples (55%) (S1 Table). The primary cause of sequencing failure was unsuccessful amplification in the second PCR of the nested PCR process. Although degradation of viral RNA during the transit time of wastewater in sewer lines are limited [37], RNA degradation might occur during the freeze-thaw process of the samples. The wastewater samples from Hamamatsu City were stored at -20°C and processed by PEG precipitation after thawing, while those from Komatsu City were concentrated by PEG precipitation before freezing at -20°C. This likely contributed to the lower sequencing success rate in samples from Hamamatsu samples (8/18, 44%) compared to Komatsu samples (13/20, 65%). Moreover, RNA degradation due to sample storage was found to be dependent on the initial viral RNA concentration rather than duration of storage and followed the first-order reaction kinetic (S4 Figure). These findings suggest that viral RNA degradation might be facilitated by the thawing process, which caused bacterial cell destruction and release of RNase from the bacterial cells. In addition to the possible degradation of viral RNA, presence of PCR inhibiting substances can also lead to amplification failure, particularly when suspended solids were not excluded from the sample [12]. Therefore, improving sample storage conditions and excluding suspended solids in the virus concentration step would enhance the reliability of detailed mutation analysis in wastewater genomic surveillance.

**Reference**

31.	Karthikeyan, Smruthi, Joshua I. Levy, Peter de Hoff, Greg Humphrey, Amanda Birmingham, Kristen Jepsen, Sawyer Farmer, et al. 2022. “Wastewater Sequencing Reveals Early Cryptic SARS-CoV-2 Variant Transmission.” Nature 609 (7925): 101–8. https://doi.org/10.1038/s41586-022-05049-6.

32.	Rosa, G. la, D. Brandtner, G. Bonanno Ferraro, C. Veneri, P. Mancini, M. Iaconelli, L. Lucentini, et al. 2023. “Wastewater Surveillance of SARS-CoV-2 Variants in October–November 2022 in Italy: Detection of XBB.1, BA.2.75 and Rapid Spread of the BQ.1 Lineage.” Science of the Total Environment 873 (May). https://doi.org/10.1016/j.scitotenv.2023.162339.

33.	Smyth, Davida S., Monica Trujillo, Devon A. Gregory, Kristen Cheung, Anna Gao, Maddie Graham, Yue Guan, et al. 2022. “Tracking Cryptic SARS-CoV-2 Lineages Detected in NYC Wastewater.” Nature Communications 13 (1). https://doi.org/10.1038/s41467-022-28246-3.

34.	Barbé, Laure, Julien Schaeffer, Alban Besnard, Sarah Jousse, Sébastien Wurtzer, Laurent Moulin, Françoise S. le Guyader, and Marion Desdouits. 2022. “SARS-CoV-2 Whole-Genome Sequencing Using Oxford Nanopore Technology for Variant Monitoring in Wastewaters.” Frontiers in Microbiology 13 (June). https://doi.org/10.3389/fmicb.2022.889811.

35.	Child, Harry T., Paul A. O’Neill, Karen Moore, William Rowe, Hubert Denise, David Bass, Matthew J. Wade, et al. 2023. “Optimised Protocol for Monitoring SARS-CoV- 2 in Wastewater Using Reverse Complement PCR-Based Whole-Genome Sequencing.” PLoS ONE 18 (4 APRIL). https://doi.org/10.1371/journal.pone.0284211.

36.	Xu, Xiaoqing, Yu Deng, Jiahui Ding, Xiawan Zheng, Chunxiao Wang, Dou Wang, Lei Liu, et al. 2023. “Wastewater Genomic Sequencing for SARS-CoV-2 Variants Surveillance in Wastewater-Based Epidemiology Applications.” Water Research 244 (October). <https://doi.org/10.1016/j.watres.2023.120444>.

37.	Ahmed, Warish, Paul M. Bertsch, Kyle Bibby, Eiji Haramoto, Joanne Hewitt, Flavia Huygens, Pradip Gyawali, et al. 2020. “Decay of SARS-CoV-2 and Surrogate Murine Hepatitis Virus RNA in Untreated Wastewater to Inform Application in Wastewater-Based Epidemiology.” Environmental Research 191 (December): 110092. https://doi.org/10.1016/j.envres.2020.110092.

**Reviewer #2:**

*The authors have made a valuable contribution to the field of wastewater-based surveillance by highlighting the limitations of quantifying variant proportions, when it comes to tracing back the origin and transmission pathways of locally dominant variants. While wastewater-derived viral genome sequences represent a mix of multiple variants, they assume that each individual amplicon read is presumably originating from a single strain. Therefore, their insightful approach emphasizes the potential of haplotype analysis using partial viral genomes to more precisely trace the origin and transmission dynamics of SARS-CoV-2 variants. This work enhances our understanding of viral evolution in wastewater and provides a framework for more accurate epidemiological tracking.*

*However, the great potential of these findings is underserved by the lack of additional information. Here are questions that authors are invited to address and use to discuss the findings and provide additional clarifications to the study, as well as additional comments that are suggested to improve this work.*

*Minor points to be addressed*

1. *Typos to be corrected: Line 34: "which had never reported in clinical samples from Japan" should be changed to " which had never been reported in clinical samples from Japan "*

Response:

Thank you for your comment. We corrected the corresponding part.

2. *Please remove highlights (references, supplementary data, figures) for the final version.*

Response:

We have removed unnecessary highlights from the manuscript.

3. *Section "2.1. Wastewater sampling": the authors should mention the total number of samples collected.*

Response:

Total 38 samples were collected for this study. We added the number of samples in the corresponding sentence.

**L.65-66**

Total 38 wastewater samples were collected by grab sampling during the morning peak flow …

4. *Lines 145-147: "To compare with known sequences sampled in Japan, we downloaded sequences of collection year 2023 from GISAID on 13 August, 2024". The authors should mention in the MM section the number of sequences downloaded.*

Response:

Total 92,128 sequences were obtained from the GISAID database. We added the number of sequence data in the corresponding sentence.

**L.123-125**

To compare with known sequences sampled in Japan, total 92,128 sequences of collection year 2023 were downloaded from GISAID [16] on 13 August 2024.

5. *Lines 153-154: "Phylogenetic trees for the sub-variants were constructed using MEGA11 and the Neighbor- Joining method". No mention is made about the parameters used, if default options were chosen, etc. This applies to other tools mentioned and deserves revision.*

Response:

We added the description on the detailed conditions in construction of the phylogenetic trees and in the Fig.2 caption as below.

**L.131-142**

A phylogenetic tree of the sub-variants was constructed using the Neighbor-Joining method [19]. The percentage of replicate trees in which the associated taxa clustered together during the bootstrap test (500 replicates) is shown next to the branches [20]. The tree is drawn to scale, with branch lengths proportional to the evolutionary distances used to infer the phylogenetic tree. Evolutionary distances were calculated using the Maximum Composite Likelihood method [21] and are expressed as the number of base substitutions per site. All ambiguous positions were removed for each sequence pair using pairwise deletion option. The final dataset consisted of 504 aligned positions between the primers S013 and S009 [5] (S1 Figure), and included 33 sub-variants sequences along with reference sequences (GenBank MN908947.3 and OY146289.1) (S1 Appendix). Evolutionary analyses were conducted using MEGA11 [22]

**Fig 2. Caption**

**Fig 2. Evolutionary relationships of SARS-CoV-2 sub-variants detected in wastewater.** Sub-variants matching the genomic surveillance data (GISAID) of SARS-CoV-2 viruses isolated from clinical specimens in Japan are shown in **bold**, while sub-variants with no matching data are shown in *italic*. Sub-variants detected only in Hamamatsu City are shown in **blue**, and those detected only in Komatsu City are shown in **orange**. Sub-variants with stop codons or frameshifts in the sequence are shown in **red**. Branches corresponding to partitions reproduced in less than 50% of bootstrap replicates are collapsed. Bootstrap values are indicated at the branches on a 0.01 scale.

**References**

19.	Naruya Saitou and Masatoshi Nei. 1987. “The neighbor-joining method: A new method for reconstructing phylogenetic trees.” Molecular Biology and Evolution 4:406-425. https://10.1093/oxfordjournals.molbev.a040454

20.	Joseph Felsenstein. 1985. “Confidence limits on phylogenies: An approach using the bootstrap.” Evolution 39:783-791. https://10.1111/j.1558-5646.1985.tb00420.x

21.	Koichiro Tamura, Masatoshi Nei, Sudhir Kumar. 2004. “Prospects for inferring very large phylogenies by using the neighbor-joining method.” Proceedings of the National Academy of Sciences (USA) 101:11030-11035. <https://10.1073/pnas.0404206101>

22.	Koichiro Tamura, Glen Stecher and Sudhir Kumar. 2021.” MEGA 11: Molecular Evolutionary Genetics Analysis Version 11.” Molecular Biology and Evolution 38(7): 3022-3027. https://doi.org/10.1093/molbev/msab120.

6. *Figure 1: Panel C and Panel D have a number or missing data. Assuming that variants cannot suddenly disappear and stop circulating from one week to another in a sawtooth pattern, is this due to missing information, sample unavailability or no variant detected at all in those weeks?*

Response:

The blank weeks indicates missing of available sequence data due to the sequencing failure. Among the 38 wastewater samples collected, amplicon sequence data were successfully acquired from 21 samples. We added descriptions on the number of obtained sequence data and explanation in the figure caption as below.

**L.115-116**

Among the total 38 samples collected, amplicon sequence data were successfully acquired from 21 samples (S1 Table).

**Fig 1. Caption**

**Fig 1. Time-series change of SARS-CoV-2 titers and variant compositions in wastewater of the target cities.**

(A) Locations of the target cities. (B) SARS-CoV-2 RNA concentrations in wastewater of Komatsu and Hamamatsu in 2023, as gene copies (gc) per liter of wastewater. (C) Proportion of SARS-CoV-2 variants by PANGO lineage in Komatsu. (D) Proportion of SARS-CoV-2 variants by PANGO lineage in Hamamatsu. The blank weeks in the proportion of SARS-CoV-2 variants indicate that no sequence data were obtained for the corresponding week.

*Major points to be addressed*

7. *Overall, the work done deserves better data interpretation and additional clarifications on the findings. Authors are invited to provide additional content to improve the manuscript readability and understanding (no reminder in the results section about the number samples sequenced, no discussion of data obtained as compared to results from other teams worldwide, etc).*

Response:

We appreciate very much for your comment. Regarding this and other comments from the reviewers, we have made substantial improvement of the manuscript including enhancement in discussion. The major changes in discussion were made in the part of:

- Regional difference in sub-variants and their epidemic durations from relations with clinical variants (L.227-241, L.262-273)
- Detection of nonsense mutations and its significance in understanding the evolutionary trajectory of the virus (L.293-301, L.294-345)
- Benefits and utility in public health and limitations of this study (L.303-371)

Reminder of the sample size and comparison with other studies have been also added as below.

*<Reminder of the sample size>*

**L.176-178**

Nucleotide-level mutation analysis of the 21 amplicon sequences from wastewater identified the presence of locally predominant sub-variants and their epidemic durations in each city.

*<Comparison with other studies on occurrence of nonsense mutations>*

**L.256-269**

In genomic studies of clinical samples, Omicron lineages have been reported to exhibit a higher frequency of nonsense mutations, including frameshifts and stop codons, compared to the Alpha, Beta, and Delta lineages, suggesting that these mutations may facilitate the adaptation of the variant. [27–29]. While the role of nonsense mutations in viral adaptation remains unclear, their higher prevalence in Omicron suggests they may be more tolerated or result from the variant’s rapid evolution [28]. Detection of nonsense mutations, such as frameshifts and stop codons, in SARS-CoV-2 genomic sequences in wastewater has been reported in a previous study [30]. However, such nonsense mutations are rarely identified by clinical genomic surveillance because clinical genomic surveillance primarily relies on samples from symptomatic and diagnosed patients only. In this study, we demonstrate for the first time the potential for elucidating the mechanisms driving the transition of epidemic lineages due to the emergence of frameshifts and stop codons through nucleotide-level monitoring of Omicron sub-lineages.

*<Comparison with other studies on utility of wastewater genomic surveillance>*

**L.283-288**

Recent studies reported that wastewater genomic surveillance could detect emerging variants earlier than through clinical surveillance [5, 31–32]. Moreover, deep sequencing of the RBD region detected cryptic SARS-CoV-2 lineages that were not found in any global databases [33]. The findings of this study also demonstrate that the utility of wastewater genomic surveillance for detection of SARS-CoV-2 sub-variants that have been uncovered by clinical surveillance.

**References**

27.	Basheer, Atia, Imran Zahoor, and Tahir Yaqub. 2023. “Genomic Architecture and Evolutionary Relationship of BA.2.75: A Centaurus Subvariant of Omicron SARS-CoV-2.” PLoS ONE 18 (5 May). https://doi.org/10.1371/journal.pone.0281159.

28.	Campos, Carolina, Sergi Colomer-Castell, Damir Garcia-Cehic, Josep Gregori, Cristina Andrés, Maria Piñana, Alejandra González-Sánchez, et al. 2022. “The Frequency of Defective Genomes in Omicron Differs from That of the Alpha, Beta and Delta Variants.” Scientific Reports 12 (1). https://doi.org/10.1038/s41598-022-24918-8.

29.	Chrysostomou, Andreas C., Bram Vrancken, Christos Haralambous, Maria Alexandrou, Ioanna Gregoriou, Marios Ioannides, Costakis Ioannou, et al. 2023. “Unraveling the Dynamics of Omicron (BA.1, BA.2, and BA.5) Waves and Emergence of the Deltacton Variant: Genomic Epidemiology of the SARS-CoV-2 Epidemic in Cyprus (Oct 2021–Oct 2022).” Viruses 15 (9). https://doi.org/10.3390/v15091933.

30.	Napit, Rajindra, Prajwol Manandhar, Ashok Chaudhary, Bishwo Shrestha, Ajit Poudel, Roji Raut, Saman Pradhan, et al. 2023. “Rapid Genomic Surveillance of SARS-CoV-2 in a Dense Urban Community of Kathmandu Valley Using Sewage Samples.” PLoS ONE 18 (3 March). <https://doi.org/10.1371/journal.pone.0283664>.

31.	Karthikeyan, Smruthi, Joshua I. Levy, Peter de Hoff, Greg Humphrey, Amanda Birmingham, Kristen Jepsen, Sawyer Farmer, et al. 2022. “Wastewater Sequencing Reveals Early Cryptic SARS-CoV-2 Variant Transmission.” Nature 609 (7925): 101–8. https://doi.org/10.1038/s41586-022-05049-6.

32.	Rosa, G. la, D. Brandtner, G. Bonanno Ferraro, C. Veneri, P. Mancini, M. Iaconelli, L. Lucentini, et al. 2023. “Wastewater Surveillance of SARS-CoV-2 Variants in October–November 2022 in Italy: Detection of XBB.1, BA.2.75 and Rapid Spread of the BQ.1 Lineage.” Science of the Total Environment 873 (May). https://doi.org/10.1016/j.scitotenv.2023.162339.

33.	Smyth, Davida S., Monica Trujillo, Devon A. Gregory, Kristen Cheung, Anna Gao, Maddie Graham, Yue Guan, et al. 2022. “Tracking Cryptic SARS-CoV-2 Lineages Detected in NYC Wastewater.” Nature Communications 13 (1). https://doi.org/10.1038/s41467-022-28246-3.

8. *Authors have not mentioned the clinical cases reported at the same period of time the study was conducted within the two geographical areas analyzed, nor they have correlated their data with the clinical cases. This would help valorize their results and could give insights into how this approach could help detect variants before their clinical characterization.*

Response:

Thank you for your comment. This study does not focus on comparison of viral titers in wastewater with clinical cases, because a number of studies had already clarified its effectiveness and limitations.

Moreover, during the study period, Japan had already discontinued reporting the total number of clinical cases in each city. Instead, the only available clinical case data were the weekly case counts across entire prefectures from selected monitoring hospitals. This data did not accurately represent the outbreak situations in the target cities but was rather influenced by outbreaks in other larger cities. (The population of Komatsu City accounts for only 9.6% of Ishikawa Prefecture; that of Hamamatsu City accounts for 22% of Shizuoka Prefecture.)

For the above reasons, we did not include discussion on that point because we have thought we cannot lead meaningful findings from those data. Here we present the SARS-CoV-2 titer in the target WWTPs and weekly case counts per monitoring hospitals in entire prefectures for your reviewing purpose. The figures below indicate that available clinical case data are no longer represent the epidemic situation at city level. We can present these charts in the manuscript if the reviewer think it is meaningful.

**Figure R1.** SARS-CoV-2 titer in the target WWTPs in Komatsu City (a) and Hamamatsu City (b), compared with weekly case counts per monitoring hospitals across entire prefectures (NIID, 2023). The number of monitoring hospitals in Ishikawa Prefecture, where Komatsu City locates, and Shizuoka Prefecture, where Hamamatsu City locates, are 48 and 138 hospitals, respectively.

**Date Source** (for the clinical data)

National Institute of Infectious Diseases (NIID) (2023) Diseases monitored by designated points (weekly reports), cumulative reports, cumulative reports per designated point, by prefecture. IDWR Flash Data Week 52, 2023, <https://www.niid.go.jp/niid/ja/data/12442-idwr-sokuho-data-j-2352.html>, Accessed on March 23, 2025

9. *Authors should report the normalization method used if any, or correlate their data to known normalization options (please refer to Figure 1, Panel B).*

Response:

In Figure 1, we simply showed viral titers in the detected gene copies per liter of the wastewater sample. We did not perform any normalization by population nor detection efficiency. The purpose of indicating the Fig. 1b is to compare predominant variants with epidemic trends by wastewater data. We added the following description in the figure caption.

**Fig 1. Caption**

**Fig 1. Time-series change of SARS-CoV-2 titers and variant compositions in wastewater of the target cities.**

(A) Locations of the target cities. (B) SARS-CoV-2 RNA concentrations in wastewater of Komatsu and Hamamatsu in 2023, as gene copies (gc) per liter of wastewater. (C) Proportion of SARS-CoV-2 variants by PANGO lineage in Komatsu. (D) Proportion of SARS-CoV-2 variants by PANGO lineage in Hamamatsu. The blank weeks in the proportion of SARS-CoV-2 variants indicate that no sequence data were obtained for the corresponding week.

10. *Authors should mention if factors such as environmental factors (viral RNA degradation, sample collection, or processing biases) could affect the reliability of haplotype analysis?*

Response:

As responded above to the comment #1-6, factors that might affect reliability of this analysis include: (i) timing of sample collection, (ii) RNA degradation during sample storage and processing, and (iii) presence of PCR inhibiting substances in wastewater.

(i) Timing of sample collection

In this study, some samples were collected by grab sampling at the morning peak-flow time. Therefore, viral RNA discharged in another time were not included in the sample. Composite sampling has better representativeness than the grab sampling; however, the representativeness depends on sampling frequence withing a day. In the composite sampling of this study, sampling was conducted with 30-min intervals, which might overlook viral RNA discharged within the sampling intervals. This is a general limitation of WBE for both quantification of viral titers and sequencing analyses.

(ii) RNA degradation during sample storage and processing

Degradation of RNA during the transit time is expected limited (<3% in 3 hours at 25°C in untreated wastewater) according to Ahmed et al. 2020, since the transit time in sewer lines of the target WWTPs were a couple of hours. However, sequence data was successfully obtained in only 21 of 38 samples (55%) (S1 Table). Samples from Hamamatsu exhibited a lower success ratio (8/18, 44%) than those from Komatsu (13/20, 65%). The sequencing failure in this study was mainly due to failure of amplification in the second PCR of the nested PCR process.

One possible reason for the sequencing failure was probably RNA degradation due to the freeze-thaw process of the samples. Wastewater samples from Hamamatsu City were stored at -20°C then processed by PEG precipitation after thawing, while those from Komatsu City were stored at -20°C after concentration by PEG precipitation. Hence, timing of freezing samples would impact the success of the sequencing analysis.

For Komatsu samples, we also examined effects of sample storage by comparing viral titers before and after storage. According to the comparison, the degradation rates of the viral titers were dependent on the initial viral RNA concentration rather than duration of storage and followed the first-order reaction kinetic (S4 Figure).

A possible mechanistic explanation for these facts is that thawing caused bacterial cell destruction and release of RNase from the bacterial cells, which facilitates degradation of viral RNA in the sample. The success ratios in Komatsu samples were better probably because cell destruction was minimized within PEG precipitates, while more RNase was released in Hamamatsu samples. This can also explain the inconsistency between the obtained RNA copies in the extract and the sequencing success ratio. Quantification of SARS-CoV-2 RNA was performed with CDCN1 assay, which targets the shorter region with 73bp, while sequencing analysis targeted the longer region with 621bp. Hence, the target region of the sequencing analysis was fragmentated more easily and subjected to amplification failure.

(iii) Presence of PCR inhibiting substances in wastewater

Another possible for the sequencing failure was presence of PCR inhibiting substances in the samples. Although degradation ratios depended on the initial viral titer, the sequencing success ratios did not depend on the obtained RNA copies in the extract. In this study, suspended solids were not excluded in the RNA extraction process. Past studies reported that viral titers tend to be fluctuated when suspended solids are included, suggesting an RNA extract from suspended solids contains more PCR inhibiting substances than that from water phase only (Alamin et al. 2022).

We added this discussion in the revised manuscript as below. We also added description on sample storage conditions in the method section and more detailed information of the sample conditions and sequencing reads as S1 Table.

**L.65-71 (Methods)**

Total 38 wastewater samples were collected by grab sampling during the morning peak flow in Komatsu and in the 33^rd^ week in Hamamatsu, and by 24-hr equal-volume composite sampling at 30-min intervals in the other weeks in Hamamatsu (S1 Table). Sampling was conducted twice monthly from February to November, 2023. Samples in Hamamatsu were stored at -20°C after sampling pending subsequent analysis, while samples in Komatsu were subjected to the subsequent analysis within 24 hours of sampling.

**L.73-77 (Methods)**

Prior to RNA extraction, 40 mL of each sample was concentrated via polyethylene glycol (PEG) precipitation without separating suspended solids [12]. Samples in Komatsu were stored at -20°C after the PEG precipitation until the subsequent viral RNA extraction. Viral RNA was extracted from the PEG-precipitated samples using the COPMAN DNA/RNA extraction kit for wastewater (AdvanSentinel, Tokyo) [13].

**L.316-341**

Despite these advantages, several limitations of wastewater genomic surveillance should be noted. The representativeness of wastewater genomic surveillance primarily depends on the sampling methodology. Grab sampling, often conducted during morning peak flow, may fail to detect variants discharged at other times. Composite sampling, which collects multiple samples over a 24-hour period, improves representativeness but may still miss variants released between sampling intervals. Additionally, improving sample storage and processing methods has been suggested to enhance the reliability of mutation analysis in wastewater genomic surveillance. In this study, sequence data was successfully obtained in only 21 of 38 samples (55%) (S1 Table). The primary cause of sequencing failure was unsuccessful amplification in the second PCR of the nested PCR process. Although degradation of viral RNA during the transit time of wastewater in sewer lines are limited [37], RNA degradation might occur during the freeze-thaw process of the samples. The wastewater samples from Hamamatsu City were stored at -20°C and processed by PEG precipitation after thawing, while those from Komatsu City were concentrated by PEG precipitation before freezing at -20°C. This likely contributed to the lower sequencing success rate in samples from Hamamatsu samples (8/18, 44%) compared to Komatsu samples (13/20, 65%). Moreover, RNA degradation due to sample storage was found to be dependent on the initial viral RNA concentration rather than duration of storage and followed the first-order reaction kinetic (S4 Figure). These findings suggest that viral RNA degradation might be facilitated by the thawing process, which caused bacterial cell destruction and release of RNase from the bacterial cells. In addition to the possible degradation of viral RNA, presence of PCR inhibiting substances can also lead to amplification failure, particularly when suspended solids were not excluded from the sample [12]. Therefore, improving sample storage conditions and excluding suspended solids in the virus concentration step would enhance the reliability of detailed mutation analysis in wastewater genomic surveillance.

**S4 Fig. Reduction of viral RNA concentration before and after sample storage.** (A) Log reduction of SARS-CoV-2 by the initial concentration. (B) Log reduction of PMMoV by the initial concentration. (C) Log reduction of SARS-CoV-2 by duration of storage. (D) Log reduction of PMMoV by duration of storage. The SARS-CoV-2 RNA concentrations were quantified with CDCN1 assay.

**Reference**

37.	Ahmed, Warish, Paul M. Bertsch, Kyle Bibby, Eiji Haramoto, Joanne Hewitt, Flavia Huygens, Pradip Gyawali, et al. 2020. “Decay of SARS-CoV-2 and Surrogate Murine Hepatitis Virus RNA in Untreated Wastewater to Inform Application in Wastewater-Based Epidemiology.” Environmental Research 191 (December): 110092. https://doi.org/10.1016/j.envres.2020.110092.

11. *Authors should discuss if integrating multiple approaches is feasible and/or could improve the accuracy of tracking viral variants in wastewater.*

Response:

In this study, haplotype analysis was applied to a short region within the receptor-binding domain (RBD) of the spike (S) protein. Recently, however, various methods have been developed for analyzing SARS-CoV-2 genome sequences in wastewater, enabling the sequencing of whole genome regions (Barbe et al. 2022; Child et al. 2023; Xu et al. 2023). Applying haplotype analysis to whole-genome sequences obtained from wastewater is expected to reveal a broader trajectory of mutations.

We added this description in the revised manuscript as below.

**L.291-297**

In this study, haplotype analysis was applied to a short region within the RBD of the S gene. However, recent advancements in sequencing techniques have enabled the analysis of whole genome sequences of SARS-CoV-2 from wastewater [34–36]. Applying haplotype analysis to whole-genome sequences obtained from wastewater is expected to provide a more comprehensive view of mutation trajectories, enhancing our understanding of viral evolution, including the emergence of nonsense mutations and asymptomatic variants.

**References**

34.	Barbé, Laure, Julien Schaeffer, Alban Besnard, Sarah Jousse, Sébastien Wurtzer, Laurent Moulin, Françoise S. le Guyader, and Marion Desdouits. 2022. “SARS-CoV-2 Whole-Genome Sequencing Using Oxford Nanopore Technology for Variant Monitoring in Wastewaters.” Frontiers in Microbiology 13 (June). https://doi.org/10.3389/fmicb.2022.889811.

35.	Child, Harry T., Paul A. O’Neill, Karen Moore, William Rowe, Hubert Denise, David Bass, Matthew J. Wade, et al. 2023. “Optimised Protocol for Monitoring SARS-CoV- 2 in Wastewater Using Reverse Complement PCR-Based Whole-Genome Sequencing.” PLoS ONE 18 (4 APRIL). https://doi.org/10.1371/journal.pone.0284211.

36.	Xu, Xiaoqing, Yu Deng, Jiahui Ding, Xiawan Zheng, Chunxiao Wang, Dou Wang, Lei Liu, et al. 2023. “Wastewater Genomic Sequencing for SARS-CoV-2 Variants Surveillance in Wastewater-Based Epidemiology Applications.” Water Research 244 (October). https://doi.org/10.1016/j.watres.2023.120444.

12. *How does haplotype analysis compare with other existing methods, such as whole-genome sequencing or computational deconvolution of mixed variants?*

Response:

Various methods have been developed and applied to analysis of SARS-CoV-2 variants in wastewater so far (Karthikeyan et al. 2022; La Rosa et al. 2023; Iwamoto et al. 2023; Barbe et al. 2022; Child et al. 2023; Xu et al. 2023). Most of the existing methods for wastewater-based genomic epidemiology (WBGE) primarily aimed to estimate proportions of variants in a community. In these studies, the existing variants (or their proportions) were identified by alignment of the sequence with the catalog of known variants. The critical difference of this haplotype analysis from other methodology is to reveal the evolutional trajectory of SARS-CoV-2.

If genetic mutations occur randomly, not all mutations are necessarily beneficial for viral replication; they also include deleterious mutations and neutral mutations, which are neither harmful nor beneficial. Strains carrying deleterious mutations are eliminated through selection, whereas those with neutral mutations persist and contribute to the formation of diverse sub-variants in different regions. On the other hand, variants recognized in clinical settings primarily possess mutations that enhance infectivity in humans (i.e., beneficial for replication). Consequently, both clinical and wastewater genomic surveillance that relies on existing catalogs of known clinical variants faces challenges in capturing the evolutionary pathways leading to the emergence of novel viral lineages. In the haplotype analysis of this study, we analyzed mutations at the nucleotide level, including synonymous substitutions, rather than focusing solely on amino acid substitutions. This approach allows for a more comprehensive understanding of viral variant diversity, including neutral mutations, and represents a novel and advantageous method compared to conventional approaches. Through this, we can elucidate the evolutionary dynamics of the virus and the detailed evolutionary trajectories leading to the emergence of new clinical variants.

We added this discussion in the revised manuscript as below.

**L.291-314**

In this study, haplotype analysis was applied to a short region within the RBD of the S gene. However, recent advancements in sequencing techniques have enabled the analysis of whole genome sequences of SARS-CoV-2 from wastewater [34–36]. Applying haplotype analysis to whole-genome sequences obtained from wastewater is expected to provide a more comprehensive view of mutation trajectories, enhancing our understanding of viral evolution, including the emergence of nonsense mutations and asymptomatic variants.

Since genetic mutations occur randomly, not all mutations are beneficial for viral replication; they also include deleterious and neutral mutations, the latter of which are neither advantageous nor disadvantageous. Strains carrying deleterious mutations are eliminated through selection, whereas those with neutral mutations persist and contribute to the formation of diverse sub-variants in different regions. In contrast, variants identified in clinical settings primarily possess mutations that enhance human infectivity (i.e., beneficial for replication). Consequently, both clinical and wastewater genomic surveillance that rely on existing catalogs of known clinical variants face challenges in capturing the evolutionary pathways leading to the emergence of novel viral lineages. To address this limitation, this study employed a nucleotide-level mutation analysis, including synonymous substitutions, rather than focusing solely on amino acid changes. This approach enables a more comprehensive understanding of viral variant diversity, particularly neutral mutations, and represents a novel and advantageous method compared to conventional approaches. By incorporating such analyses, we can better elucidate the evolutionary dynamics of the virus and the detailed trajectories leading to the emergence of new clinical variants.

**References**

34.	Barbé, Laure, Julien Schaeffer, Alban Besnard, Sarah Jousse, Sébastien Wurtzer, Laurent Moulin, Françoise S. le Guyader, and Marion Desdouits. 2022. “SARS-CoV-2 Whole-Genome Sequencing Using Oxford Nanopore Technology for Variant Monitoring in Wastewaters.” Frontiers in Microbiology 13 (June). https://doi.org/10.3389/fmicb.2022.889811.

35.	Child, Harry T., Paul A. O’Neill, Karen Moore, William Rowe, Hubert Denise, David Bass, Matthew J. Wade, et al. 2023. “Optimised Protocol for Monitoring SARS-CoV- 2 in Wastewater Using Reverse Complement PCR-Based Whole-Genome Sequencing.” PLoS ONE 18 (4 APRIL). https://doi.org/10.1371/journal.pone.0284211.

36.	Xu, Xiaoqing, Yu Deng, Jiahui Ding, Xiawan Zheng, Chunxiao Wang, Dou Wang, Lei Liu, et al. 2023. “Wastewater Genomic Sequencing for SARS-CoV-2 Variants Surveillance in Wastewater-Based Epidemiology Applications.” Water Research 244 (October). https://doi.org/10.1016/j.watres.2023.120444.

13. *The study assumes that each amplicon read originates from a single strain. However, mixed infections and recombination events could complicate this assumption. Authors should highlight how they assess the accuracy of this assumption. Does the method provide enough resolution to differentiate between closely related variants?*

Response:

We appreciate for your comment. Strictly speaking, each amplicon is not necessarily originated from the single strain. We have toned down the corresponding text in the introduction as below.

We think your concerns on mixed infection and recombination do not affect our finding for the below reasons.

1. Impacts of mixed infection

Simultaneous infection of multiple variants to a single person is reportedly rare at 0.25~0.35% of all infections (Pipek et al. 2024; Tremeaux et al. 2023). In such cases, viral genetic elements of two or more variants would be shed and detected in wastewater. Considering the low probability of multiple variant infection, its impacts on the estimated proportion of variants by WBE would be much smaller than the possible fluctuation of viral shedding among population.

2. Possible impacts of recombination and amplification errors

From our careful examination of the obtained sequence data, recombination during sequencing analysis did not occur. Recombination found in our sequence data was all consistent with the known recombination in the GISAID database. PCR amplifications were more likely to happen compared to recombination. The maximum error rate of the applied sequencing methodology was reported as 2% (Kuroiwa et al., 2023). Therefore, we exclude the mutations which occurred at lower rates than the above-mentioned maximum error rate. All the nonsense mutations presented in this study were observed at the significantly higher rate than the amplification errors, and we are confident their occurrence.

**References:**

- Pipek, Orsolya Anna, Anna Medgyes-Horváth, József Stéger, Krisztián Papp, Dávid Visontai, Marion Koopmans, David Nieuwenhuijse, et al. 2024. “Systematic Detection of Co-Infection and Intra-Host Recombination in More than 2 Million Global SARS-CoV-2 Samples.” Nature Communications 15 (1). https://doi.org/10.1038/s41467-023-43391-z.
- Trémeaux, Pauline, Justine Latour, Noémie Ranger, Vénicia Ferrer, Agnès Harter, Romain Carcenac, Pauline Boyer, et al. 2023. “SARS-CoV-2 Co-Infections and Recombinations Identified by Long-Read Single-Molecule Real-Time Sequencing.” Microbiology Spectrum 11 (4). https://doi.org/10.1128/spectrum.00493-23.

**L.47-51**

Although viral genome sequences in wastewater originate from multiple variants, the proportion of amplicon reads is assumed to reflect the relative abundance of these variants. Hence, by detailed comparison of single-nucleotide variants (SNVs) with haplotype data of clinical genome surveillance, the origin and behaviors of the locally predominant SARS-CoV-2 variants could be traced more precisely.

14. *Can this approach be applied universally, or are there constraints (sequencing depth, bioinformatics tools, computational requirements, etc) that may limit its implementation? As an example, how feasible is this method in low-resource settings?*

Response:

The primary purpose of this scientific study is to demonstrate that application of haplotype analysis to WBE enables to clarify regional dissemination pathway and local evolutionary processes of variants. Although this study does not primarily aim to prove the direct utility to public health polices, we assume the analysis we performed is potentially applied to public health policies, e.g., early warning on emergence of a new variant. These benefits were summarized in the revised manuscript as below.

Although resource settings in LMICs are quite different among countries, the haplotype analysis applied in this study requires no special resources, compared to the standard amplicon sequencing analysis of SARS-CoV-2. Illumina MiSeq System was used for the sequencing. The sequencing depth is in a standard range (20,000–35,000 raw read pairs) (S1 Table). Sequence data analysis tools used in this study are all freewares. The heaviest computational processes were phylogenetic clustering, which required less than 1GB memory. Information of the sequence depth is provided in the revised manuscript as S1 Table.

15. *In terms of public health impact, how does this method improve real-time variant tracking for public health decision-making? Could it be integrated into routine wastewater surveillance programs for early warning of emerging variants?*

Response:

We appreciate for your comment. As responded to #1-5, the primary purpose of this scientific study is to demonstrate that application of haplotype analysis to wastewater genomic surveillance enables to clarify regional dissemination pathway and local evolutionary processes of variants. One important benefit in application of the haplotype analysis to wastewater genomic surveillance is to identify mutations in a population more comprehensively than clinical genomic surveillance. For example, nonsense mutations such as frameshifts and stop codons were detected in this study as well as a past study on wastewater genomic surveillance (Napit et al. 2023). Such nonsense mutations are hardly identified by clinical genomic surveillance, which targets samples from symptomatic patients. This study demonstrated that the haplotype analysis of wastewater data could reveal virus’s evolutionary trajectory, including the emergence of nonsense mutations like frameshifts and stop codons, which would be overlooked by clinical genomic surveillance.

Although this study does not primarily aim to prove the direct utility to public health polices, we assume the analysis we performed is potentially applied to public health policies, e.g., early warning on emergence of a new variant.

In general, benefits of wastewater genomic surveillance compared to clinical genomic surveillance include:

- More efficient and frequent surveillance (e.g., on a weekly basis) due to the smaller number of samples required.
- Better representativeness of the actual proportions of variants in a target area, as it is less affected by sampling biases.
- A lower likelihood of overlooking epidemic variants due to reduced sampling biases.

In addition to these general benefits, the application of haplotype analysis would enable:

- Early detection of the emergence of local variants.
- Identification of the origins and dissemination pathways at both national and regional levels.
- Insights into the evolutionary processes of the virus, including the emergence of asymptomatic variants.

We added this discussion in the revised manuscript as below.

**L.262-272**

Detection of nonsense mutations, such as frameshifts and stop codons, in SARS-CoV-2 genomic sequences in wastewater has been reported in a previous study [30]. However, such nonsense mutations are rarely identified by clinical genomic surveillance because clinical genomic surveillance primarily relies on samples from symptomatic and diagnosed patients only. In this study, we demonstrate for the first time the potential for elucidating the mechanisms driving the transition of epidemic lineages due to the emergence of frameshifts and stop codons through nucleotide-level monitoring of Omicron sub-lineages. Furthermore, applying haplotype analysis to wastewater-based epidemiology (WBE) enables effective detection of these mutations, providing insights into more detailed evolutionary trajectories of the virus.

**L.277-297**

Compared to clinical genomic surveillance, wastewater genomic surveillance offers several advantages: (i) more efficient and frequent surveillance (e.g., on a weekly basis) due to the smaller number of samples required; (ii) better representativeness of the actual proportions of variants in a target area; and (iii) a lower likelihood of overlooking epidemic variants. These benefits complement clinical genomic surveillance, which requires a large number of samples and is subject to significant biases in sample selection. Recent studies reported that wastewater genomic surveillance could detect emerging variants earlier than through clinical surveillance [5, 31–32]. Moreover, deep sequencing of the RBD region detected cryptic SARS-CoV-2 lineages that were not found in any global databases [33]. The findings of this study also demonstrate that the utility of wastewater genomic surveillance for detection of SARS-CoV-2 sub-variants that have been uncovered by clinical surveillance. Furthermore, detailed mutation analysis in wastewater genomic surveillance facilitates the early detection of emerging local variants and the identification of their origins and dissemination pathways by comparing them with those in other countries and regions. In this study, haplotype analysis was applied to a short region within the RBD of the S gene. However, recent advancements in sequencing techniques have enabled the analysis of whole genome sequences of SARS-CoV-2 from wastewater [34–36]. Applying haplotype analysis to whole-genome sequences obtained from wastewater is expected to provide a more comprehensive view of mutation trajectories, enhancing our understanding of viral evolution, including the emergence of nonsense mutations and asymptomatic variants.

**Reference**

30.	Napit, Rajindra, Prajwol Manandhar, Ashok Chaudhary, Bishwo Shrestha, Ajit Poudel, Roji Raut, Saman Pradhan, et al. 2023. “Rapid Genomic Surveillance of SARS-CoV-2 in a Dense Urban Community of Kathmandu Valley Using Sewage Samples.” PLoS ONE 18 (3 March). <https://doi.org/10.1371/journal.pone.0283664>.

31.	Karthikeyan, Smruthi, Joshua I. Levy, Peter de Hoff, Greg Humphrey, Amanda Birmingham, Kristen Jepsen, Sawyer Farmer, et al. 2022. “Wastewater Sequencing Reveals Early Cryptic SARS-CoV-2 Variant Transmission.” Nature 609 (7925): 101–8. https://doi.org/10.1038/s41586-022-05049-6.

32.	Rosa, G. la, D. Brandtner, G. Bonanno Ferraro, C. Veneri, P. Mancini, M. Iaconelli, L. Lucentini, et al. 2023. “Wastewater Surveillance of SARS-CoV-2 Variants in October–November 2022 in Italy: Detection of XBB.1, BA.2.75 and Rapid Spread of the BQ.1 Lineage.” Science of the Total Environment 873 (May). https://doi.org/10.1016/j.scitotenv.2023.162339.

33.	Smyth, Davida S., Monica Trujillo, Devon A. Gregory, Kristen Cheung, Anna Gao, Maddie Graham, Yue Guan, et al. 2022. “Tracking Cryptic SARS-CoV-2 Lineages Detected in NYC Wastewater.” Nature Communications 13 (1). https://doi.org/10.1038/s41467-022-28246-3.

34.	Barbé, Laure, Julien Schaeffer, Alban Besnard, Sarah Jousse, Sébastien Wurtzer, Laurent Moulin, Françoise S. le Guyader, and Marion Desdouits. 2022. “SARS-CoV-2 Whole-Genome Sequencing Using Oxford Nanopore Technology for Variant Monitoring in Wastewaters.” Frontiers in Microbiology 13 (June). https://doi.org/10.3389/fmicb.2022.889811.

35.	Child, Harry T., Paul A. O’Neill, Karen Moore, William Rowe, Hubert Denise, David Bass, Matthew J. Wade, et al. 2023. “Optimised Protocol for Monitoring SARS-CoV- 2 in Wastewater Using Reverse Complement PCR-Based Whole-Genome Sequencing.” PLoS ONE 18 (4 APRIL). https://doi.org/10.1371/journal.pone.0284211.

36.	Xu, Xiaoqing, Yu Deng, Jiahui Ding, Xiawan Zheng, Chunxiao Wang, Dou Wang, Lei Liu, et al. 2023. “Wastewater Genomic Sequencing for SARS-CoV-2 Variants Surveillance in Wastewater-Based Epidemiology Applications.” Water Research 244 (October). https://doi.org/10.1016/j.watres.2023.120444.

**Other revisions made by authors:**

1. Conclusions were added to follow the formatting instruction of the journal as required below.

**L.343-354**

**Conclusions**

Nucleotide-level mutation analysis was performed on SARS-CoV-2 genome sequences obtained from wastewater in two geographically distinct cities. Genotyping of wastewater sequence data revealed the presence of the XBT variant, which had never been reported in clinical samples from Japan. Haplotype analysis further identified locally unique sub-variants in each city, suggesting that the epidemic sub-variants in these locations are evolutionarily distinct. Notably, several sub-variants detected in wastewater carried nonsense mutations, such as frameshifts and stop codons, which were rarely identified through clinical genomic surveillance. Consequently, applying haplotype analysis to WBE is expected to elucidate detailed evolutionary trajectories, including the emergence of nonsense mutations and asymptomatic variants, and to enhance our understanding of viral evolution.

2. We have revised and provided the following additional supplementary information to support the revisions regarding the review comments.

- **S1 Appendix. FASTA-format sequences of 33 sub-variants identified in this study, along with reference sequences.**
- **S4 Figure. Reduction of viral RNA concentration before and after sample storage.** (A) Log reduction of SARS-CoV-2 by the initial concentration. (B) Log reduction of PMMoV by the initial concentration. (C) Log reduction of SARS-CoV-2 by duration of storage. (D) Log reduction of PMMoV by duration of storage. The SARS-CoV-2 RNA concentrations were quantified with CDCN1 assay.
- **S1 Table. Information of samples and sequence data.** (a) Hamamatsu City. (b) Komatsu City.
- **S4 Table. Number of clinical sub-variants reported in GISAID in the whole Japan, Ishikawa Prefecture, and Shizuoka Prefecture.**

3. We refined the terminology by distinguishing between “wastewater-based genomic epidemiology” and “wastewater genomic surveillance” throughout the manuscript to ensure accurate and consistent usage.
4. Minor revisions were made for grammar corrections and typos and to enhance readability. All the changes have been highlighted in red.

**Journal Requirements:**

*When submitting your revision, we need you to address these additional requirements.*

1. *Please ensure that your manuscript meets PLOS ONE’s style requirements, including those for file naming.*

Response:

We carefully checked the journal guidelines and formatted the manuscript accordingly. Citations in the manuscript were revised into reference numbers accordingly.

2. *Please state what role the funders took in the study. If the funders had no role, please state: “The funders had no role in study design, data collection and analysis, decision to publish, or preparation of the manuscript.” If this statement is not correct you must amend it as needed.*

*Please include this amended Role of Funder statement in your cover letter; we will change the online submission form on your behalf.*

Response:

All the funders had no role in study design, data collection and analysis, decision to publish, or preparation of the manuscript. We have stated that in cover letter.

3. *We note that you have provided funding information that is not currently declared in your Funding Statement. However, funding information should not appear in the Acknowledgments section or other areas of your manuscript. We will only publish funding information present in the Funding Statement section of the online submission form.*

*Please remove any funding-related text from the manuscript and let us know how you would like to update your Funding Statement. Currently, your Funding Statement reads as follows: [JST CREST (JPMJCR20H1), JSPS KAKENHI Grants (21KK0073), Grants by civil engineering companies of Hiramoto-Gumi Inc. and I-Tec- Muramoto Co. Ltd].*

*Please include your amended statements within your cover letter; we will change the online submission form on your behalf.*

Response:

We have deleted the funding information from the acknowledge statement in the manuscript. We would like to add Funding Statement (including one additional funding) as below. We stated in the cover letter, too. In the Acknowledgment, we added acknowledgement statement for provision of computational resources.

**Funding:** JST CREST (JPMJCR20H1), JSPS KAKENHI Grants (21KK0073), JST Program Manager (PM) Development and Promotion Program, Grants by Hiramoto-Gumi Inc. and I-Tec-Muramoto Co. Ltd.

**Acknowledgments**

The authors are thankful to Hamamatsu City and Komatsu City for their cooperation in provision of wastewater samples. Computations were partially performed on the NIG supercomputer at ROIS National Institute of Genetics.

4. *In the online submission form, you indicated that your data is available only on request from a third party. Please note that your Data Availability Statement is currently missing [the name of the third party contact or institution / contact details for the third party, such as an email address or a link to where data requests can be made]. Please update your statement with the missing information.*

Response:

DDBJ is the public sequence data repository and a member of the INSDC (International Nucleotide Sequence Database Consortium) with GenBank and ENA. I added the URL of DDBJ in the data availability statement as below.

All relevant data are available in the paper and its Supporting Information files. The amplicon sequence data were deposited in the DNA Data Bank of Japan (DDBJ) Sequence Read Archive at https://ddbj.nig.ac.jp/ (accession numbers: DRR624547-DRR624567).

5. *Please review your reference list to ensure that it is complete and correct. If you have cited papers that have been retracted, please include the rationale for doing so in the manuscript text, or remove these references and replace them with relevant current references. Any changes to the reference list should be mentioned in the rebuttal letter that accompanies your revised manuscript. If you need to cite a retracted article, indicate the article’s retracted status in the References list and also include a citation and full reference for the retraction notice.*

Response:

We have confirmed all references are not retracted and relevant.

6. *While revising your submission, please upload your figure files to the Preflight Analysis and Conversion Engine (PACE) digital diagnostic tool, https://pacev2.apexcovantage.com/. PACE helps ensure that figures meet PLOS requirements. To use PACE, you must first register as a user. Registration is free. Then, login and navigate to the UPLOAD tab, where you will find detailed instructions on how to use the tool. If you encounter any issues or have any questions when using PACE, please email PLOS at* [*figures@plos.org.*](mailto:figures@plos.org) *Please note that Supporting Information files do not need this step.*

Response:

We have checked all the figures with PACE tool and converted accordingly.
